# Physical activity and sedentary behaviour research in Thailand: a systematic scoping review

**DOI:** 10.1186/s12889-018-5643-y

**Published:** 2018-06-14

**Authors:** Nucharapon Liangruenrom, Kanyapat Suttikasem, Melinda Craike, Jason A. Bennie, Stuart J. H. Biddle, Zeljko Pedisic

**Affiliations:** 1 Institute for Health and Sport, Victoria University, PO Box 14428, Melbourne, VIC 8001 Australia; 2 Institute for Population and Social Research, Mahidol University, Phutthamonthon Sai 4 Road, Salaya, Phutthamonthon, Nakhon Pathom, 73170 Thailand; 3 Institute for Resilient Regions, University of Southern Queensland, Education City, 37 Sinnathamby Boulevard, Springfield Central, QLD 4300 Australia

**Keywords:** Physical activity, Sedentary behaviour, Scoping review, Thailand

## Abstract

**Background:**

The number of deaths per year attributed to non-communicable diseases is increasing in low- and middle-income countries, including Thailand. To facilitate the development of evidence-based public health programs and policies in Thailand, research on physical activity (PA) and sedentary behaviour (SB) is needed. The aims of this scoping review were to: (i) map all available evidence on PA and SB in Thailand; (ii) identify research gaps; and (iii) suggest directions for future research.

**Methods:**

A systematic literature search was conducted through 10 bibliographic databases. Additional articles were identified through secondary searches of reference lists, websites of relevant Thai health organisations, Google, and Google Scholar. Studies written in Thai or English were screened independently by two authors and included if they presented quantitative or qualitative data relevant to public health research on PA and/or SB.

**Results:**

Out of 25,007 screened articles, a total of 564 studies were included in the review. Most studies included PA only (80%), 6.7% included SB only, and 13.3% included both PA and SB. The most common research focus was correlates (58.9%), followed by outcomes of PA/SB (22.2%), prevalence of PA/SB (12.4%), and instrument validation (3.2%). Most PA/SB research was cross-sectional (69.3%), while interventions (19.7%) and longitudinal studies (2.8%) were less represented. Most studies (94%) used self-reports of PA/SB, and few (2.5%) used device-based measures. Both sexes were examined in most studies (82.5%). Adults were the main target population group (51.1%), followed by older adults (26.9%), adolescents (15.7%), and children (6.3%). Clinical populations were investigated in the context of PA/SB in a relatively large number of studies (15.3%), most frequently those with cardiovascular disease, diabetes, and hypertension (22%, 21%, and 21% respectively).

**Conclusions:**

The number of Thai papers on PA published per year has been increasing, indicating a growing interest in this research area. More studies using population-representative samples are needed, particularly among children and adolescents, and investigating SB as a health risk factor. To provide stronger evidence on determinants and outcomes of PA/SB, longitudinal studies using standardised measures of PA and SB are required.

**Electronic supplementary material:**

The online version of this article (10.1186/s12889-018-5643-y) contains supplementary material, which is available to authorized users.

## Background

Deaths caused by non-communicable diseases (NCDs), such as cardiovascular disease and cancer, are common worldwide. Global rates of deaths attributed to NCDs increased from 60% in 2000 to 70% in 2015 [1]. Importantly, the rates of mortality caused by NCDs are increasing faster in low- and middle-income countries than in high-income countries [1]. In Thailand, NCD mortality rates increased from 64% in 2000 to 71% in 2015 [1]. Strong evidence has shown positive impacts of physical activity (PA) on the prevention of NCDs [2–5]. Some evidence also suggests that excessive sedentary behaviour (SB) (e.g. sitting) may increase the risk of several common NCDs, independently of PA [6]. It should be noted, however, that recent methodological papers questioned the independence of PA and SB, based on the argument that these behaviours are co-dependent parts of a time-use composition [7–9]. Nevertheless, the prevalence of physical inactivity, defined as not meeting the recommended level of moderate-to-vigorous physical activity (MVPA) and excessive SB, defined as sitting or reclining with low energy expenditure for more than 7 hours/day, is still high across the world, particularly in middle- and high-income countries [10–12]. In 2012, it was estimated that nearly three-quarters of all physical inactivity-related deaths occurred in low- and middle-income countries [13]. In Thailand, it was estimated that 6.3% of total mortality cases could be attributable to physical inactivity in 2013 [14]. Although, no country-specific estimates are available for Thailand, global estimates suggest that excessive SB is responsible for 3.4% of all-cause mortality [12].

Thailand has been affected by urbanisation, where, in search of better socioeconomic opportunities, many young working people move to urban areas or cities, especially to the capital, Bangkok. According to the Department of Economic and Social Affairs, United Nations, half of the Thai population (51.1%) is urban [13]. This increased rapidly from 1955 when only 18% of the Thai population lived in urban areas [13]. Many issues have arisen as a consequence of the increasing number of people living in the urban setting. An emerging concern related to urbanisation is the increasing time spent in SB in Thai population and its negative health outcomes [14]. In Thailand, there has been increasing focus on strategies to improve engagement in PA and reduce SB. Thailand has experienced significant economic development over the past four decades, moving from a low-income to upper-middle income economy [15]. Since 2002, Thailand has established a “Universal Health Coverage” scheme, to provide healthcare and financial protection to all Thai nationals [16].

As part of the national health promotion strategies, the Thai Government has aimed to promote engagement in PA since 1997 and has recently included targets to reduce SB as ways to reduce the burden of NCDs [17]. Moreover, a number of national actions have been taken to help achieve the World Health Organization’s (WHO) 15-year global target, set in 2010, of 10% reduction in the prevalence of physical inactivity, defined as less than 60 minutes of MVPA daily for adolescents and 150 minutes of MVPA weekly for persons aged 18 and over [17, 18]. WHO has commended Thailand as the regional leader in developing national health policies to promote better health through increasing PA [19]. Many PA promoting initiatives and public campaigns were introduced in Thailand, such as the development of new cycle paths, marathons organised all over the country, and a weekly program of aerobic exercise at workplace launched and led by the Prime Minister of Thailand [17, 19, 20]. Further, the national strategies and guidelines for increasing PA and reducing SB were developed [21]. Despite initiatives to increase the Thai population’s engagement in PA, population-based studies suggest that the prevalence of physical inactivity has increased from 18.5% in 2008 [22] to 19.2% in 2014 [23]. This suggests that the development and implementation of effective public health programs and policies to promote PA and decrease SB is needed.

In PA and SB epidemiology, a number of literature reviews have been conducted. For example, reviews have examined worldwide patterns of PA and SB, and show a shift from physically active to sedentary lifestyles [24–26]. Other reviews have examined factors associated with PA and SB, and the efficacy of interventions to influence the behaviours, especially in high-income countries [27–33]. However, most previous literature reviews are restricted to English language studies only and, therefore, studies from many low- and middle-income countries, including Thailand, have typically not been included. Furthermore, many previous reviews on PA and SB are restricted to specific, narrow topics (e.g. environmental determinants of PA) [27]. A comprehensive assessment of epidemiological evidence on PA and SB in the Thai context is lacking. To provide directions for future studies informing public health policies and actions targeted to increase PA and reduce SB, it is important to map the available evidence on epidemiology of PA and SB in Thailand. Scoping reviews have shown to be a useful method for a systematic assessment of the current body of evidence in a broad subject area [34]. In this study, we conducted a systematic scoping review to assess previous Thai PA and SB research, to identify research gaps and provide evidence-based directions for future research on PA and SB in Thailand to guide the development of strategies and policies.

## Methods

### Search strategy

This scoping review was conducted according to the Guidance for Conducting Systematic Scoping Reviews [35]. It included primary and secondary database searches. The primary literature search was conducted from database inception to September 2016 through the following bibliographic databases: Academic Search Premier; CINAHL; Health Source: Nursing/Academic Edition; MasterFILE Premier; PsycINFO; PubMed/MEDLINE; Scopus; SPORTDiscus; Web of Science (including Science Citation Index Expanded, Social Sciences Citation Index, Arts & Humanities Citation Index, Conference Proceedings Citation Index- Science, and Conference Proceedings Citation Index- Social Science & Humanities); and the Networked Digital Library of Theses and Dissertations (NDLTD). PubMed/MEDLINE, Scopus and Web of Science databases were searched using their own search engines, whilst other databases were searched through EBSCOhost. The search was conducted through titles, abstracts, and keywords of the indexed publications. The detailed search strategies, including the full search syntaxes, used for each database can be found in Additional file 1.

Additional articles and grey literature documents were identified via secondary literature searching through: (i) the reference lists of all articles selected in the primary search; (ii) websites of ten relevant Thai public health institutions and organizations, including the Division of Physical Activity, Ministry of Public Health; Thai Health Promotion Foundation; Physical Activity Research Centre; Health Systems Research Institute; Thai NCD Network; Thai National Research Repository; Thai Thesis Database; and three university sources including Institute for Population and Social Research, Mahidol University; Chulalongkorn University Intellectual Repository; and Kasetsart University Research and (iii) Google and Google Scholar.

### Study selection and inclusion criteria

All references from the primary database search were imported in EndNote X7 software (Thompson Reuters, San Francisco, CA, USA). After removing duplicates, the references were screened independently by two authors (NL and KS). The discrepancies between the study selections were resolved in discussion and consensus with a third author (ZP).

Studies were included in the present review, if they: (i) targeted any population group living in Thailand; (ii) conducted research on PA, physical inactivity, and/or SB; (iii) presented any quantitative or qualitative data relevant to public health, including but not limited to the levels, prevalence, correlates, determinants, or outcomes of engagement in PA and/or SB; or described the development or performed an evaluation of a PA and/or SB measurement tool or intervention; (iv) used any type of PA and/or SB measure, such as self-reports or device-based measures; (v) were written in Thai or English; and (vi) published as a journal article, conference paper, conference abstract, Master’s thesis, Doctoral thesis, or report. Studies were excluded, if they: targeted non-Thai populations; had the primary outcome(s) focusing on sports/exercise performance, or physical therapy; and were published as literature reviews, commentaries, and editorials.

### Data extraction

The following data were extracted from the included studies: (i) general bibliographical information, including author names, publication year, title, publication type, full text availability, language of full text, abstract availability, and language of abstract; (ii) description of research methods, including study design, survey method, sample size, and sampling method; (iii) information about the study population, including sex, age, municipality (rural/urban), region, and other specific characteristics of participants; (iv) description of measures, including the type of PA/SB measure, device model or questionnaire name, domains included (such as work, transport, and leisure-time), information about whether the measure has been validated or not (if applicable), and intervention type (if applicable); and (v) information about the study objectives. The detailed data extraction table for studies used in the review is available in Additional file 2.

## Results

### Search results

**Fig. 1:**
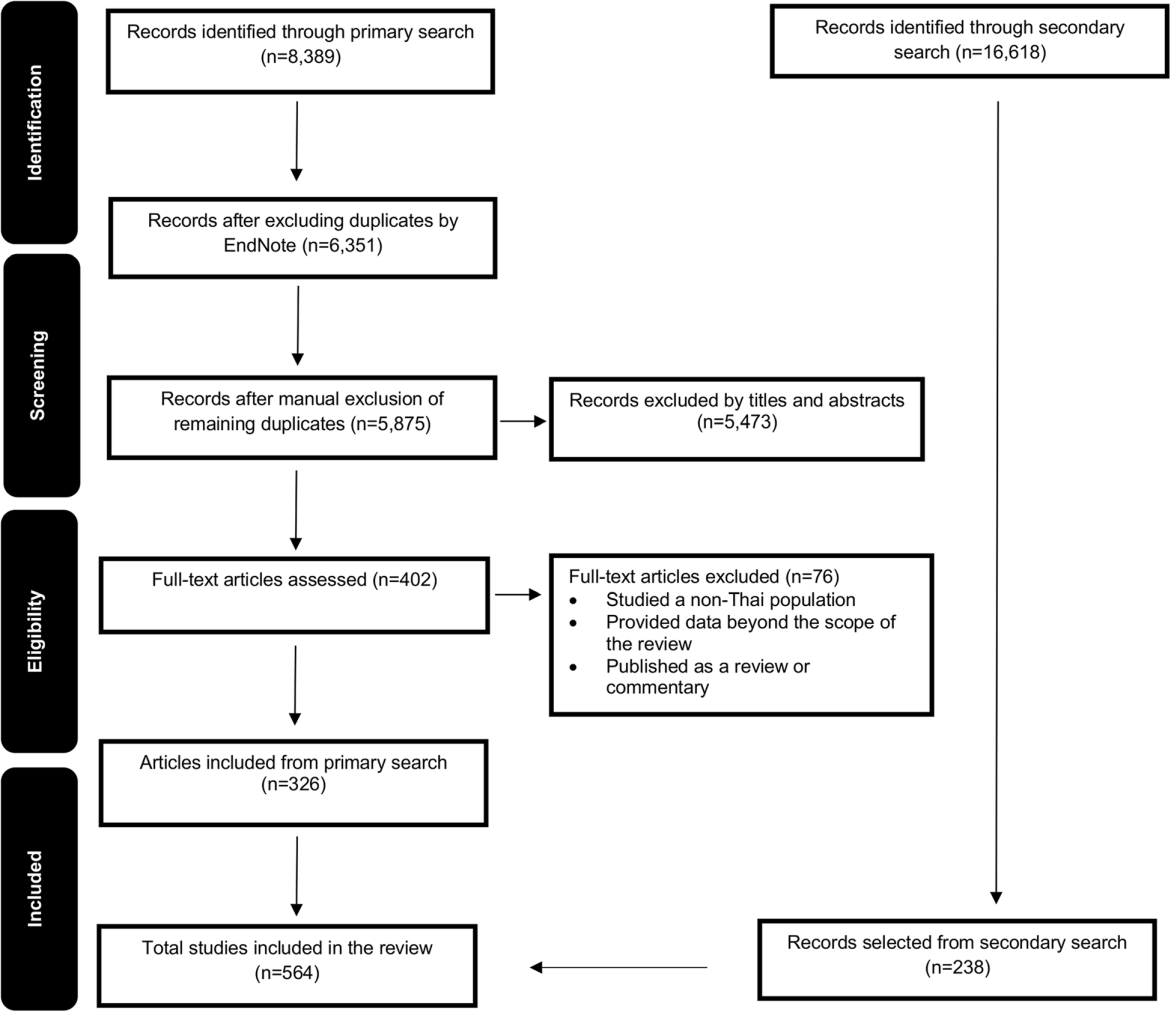
Flow diagram of study selection process

The flow diagram depicting the search and study selection processes can be found in Fig. 1. A total of 25,007 records were screened for inclusion. Of these, 8,389 studies were identified through primary searches, where, after removing duplicates, the titles and abstracts of 5,875 and full texts of 402 articles were screened. The secondary search yielded 16,618 results, of which 238 articles were selected. Overall, a total of 564 studies were included for review [36–598].

### Bibliographic characteristics of included studies

**Fig. 2:**
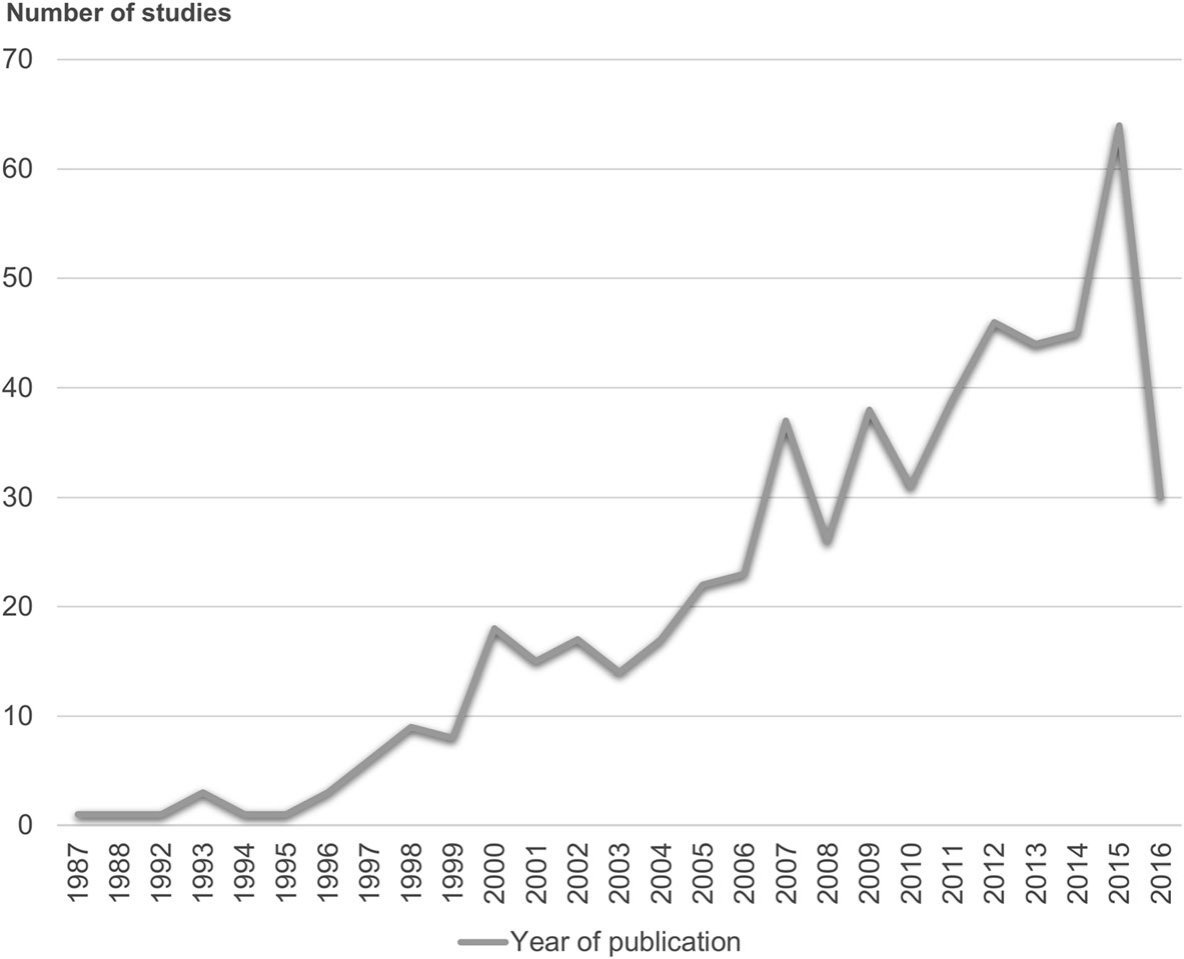
The number of Thai studies on physical activity and sedentary behaviour published per year

**Fig. 3:**
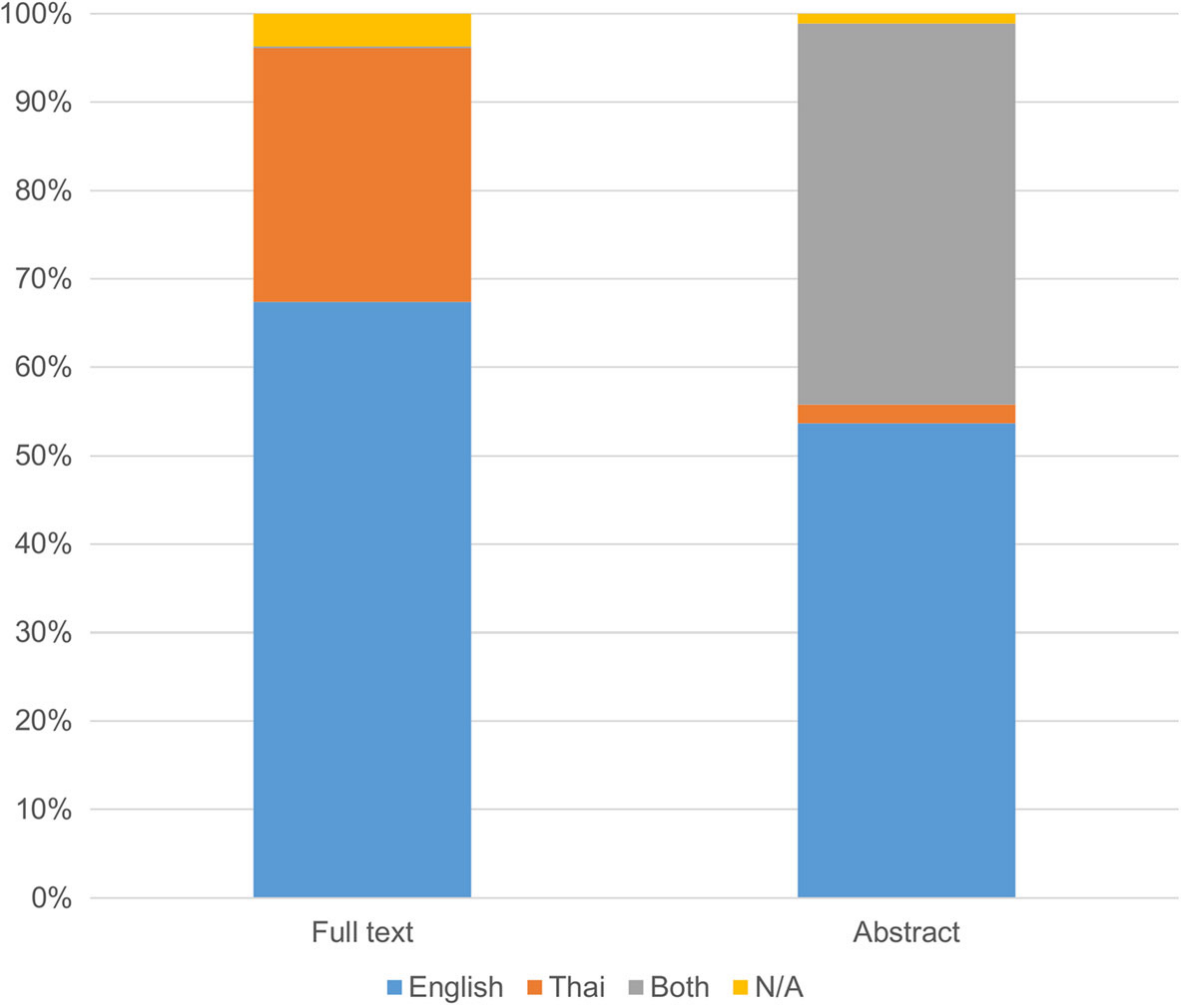
Languages used in full-texts and abstracts of Thai physical activity and sedentary behaviour publications

All papers included in this review were published between 1987 and 2016. The number of papers published per year has increased over time (Fig. 2). English was the primary language used in the majority of Thai PA/SB papers full texts (67.4%), whilst nearly all papers (*n* = 546) had at least an English abstract (Fig. 3). Furthermore, 17% of full-text articles and 10.1% of abstracts were not available online, and, therefore, other means were used to access the publications (e.g. authors’ contacts and request through university libraries). Most studies were peer-reviewed journal articles (68.3%), followed by theses (19.9%), conference papers (6.6%), and reports (5.3%).

### Study characteristics

**Fig. 4:**
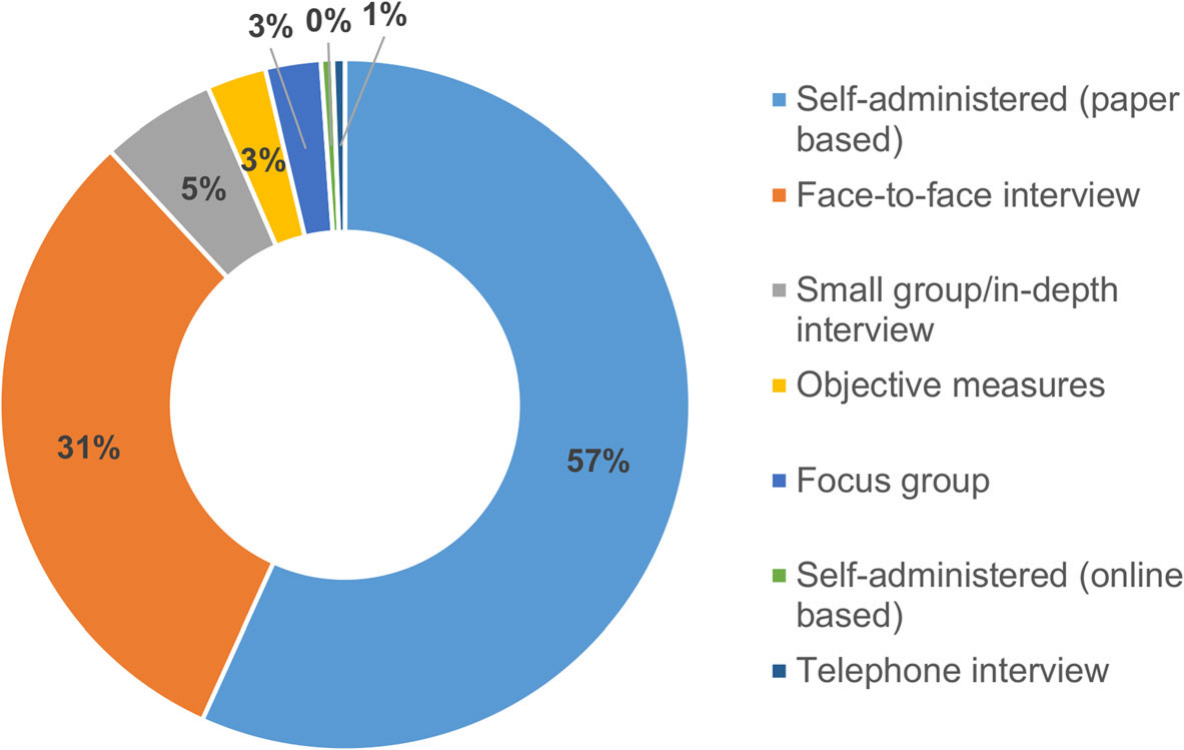
Measures of physical activity and sedentary behaviour used in Thai studies

In 363 of 564 included studies (64.4%), PA and/or SB were the primary focus of the research (e.g. a study on correlates of PA), whilst the remaining studies were not strictly focused on PA and/or SB but were analysed among multiple other variables as key explanatory or outcome variables together with PA and/or SB (e.g. other lifestyle characteristics such as smoking). Eighty percent of the studies included PA only, 6.7% included SB only, and 13.3% included both PA and SB. Most studies focused on correlates of PA/SB (58.9%), followed by outcomes of PA/SB (22.2%), prevalence of PA/SB (12.4%), and instrument validation (3.2%). 69.3% of studies used cross-sectional designs. Less represented were intervention trials (19.7%), case-control studies (3.7%), longitudinal studies (2.8%), and measurement studies (2.3%). The majority of studies used quantitative methods (87.9%), with only 4.6% and 7.5% utilising qualitative methods or mixed-methods, respectively. In most studies, the data was collected using self-administered surveys (56.7%) or face-to-face interviews (31.4%) (Fig. 4). The sample sizes of the studies ranged from 6 to 113,882 and 7.8% of the studies were conducted using nationally representative samples. Among the studies in nationally representative samples, 29.5% were secondary data analyses of the following national surveys: National Health Examination Survey; National Elderly Survey; Thailand Global School-Based Student Health Survey; 2007 National Physical Activity and Obesity Survey; and 2010 Evaluation of Health Promotion and Sports in Regions. There were seven government reports on PA and/or SB levels presenting results from population-based studies, such as the Health and Welfare Survey 2015 conducted by the National Statistical Office and National Health Examination Survey conducted by the National Health Examination Survey Office.

### Characteristics of study samples

Participants of both sexes were included in 82.5% of studies. Studies of females only (15.4%) were more common than studies of males only (2.1%). Adults (18-59 years) were the most frequently investigated age group (51.1%), followed by older adults (60+ years; 26.9%), adolescents (10 to 17 years; 15.7%), children (4 to 9 years; 5.4%), and infants/toddlers (0 to 3 years; 0.9%). A large majority of studies were conducted in non-clinical populations (84.7%). Of these, 28.5% were conducted among primary-school, secondary-school, high-school, and university students. Employees in health-related professions, including nurses, physicians, and health-care students such as medical residents were participants in 9.8% of studies. Other specific occupations were represented in 6.5% of studies; most common among them were farmers, military personnel, university staff, and office workers. Some studies (2.1%) were conducted among employees in specific organizations, such as the Electricity Generating Authority of Thailand, Metropolitan Waterworks, and the Teachers Council. Other specific non-clinical populations included in the studies were, for instance, people with low or high level of PA or SB regularity (5.6%), obese/overweight people (3.8%), women before or in menopause (2.9%), pregnant women (1.3%), and tobacco smokers (0.4%). Clinical populations were also examined in the context of PA/SB (17.7%). Patients with cardiovascular disease, diabetes, and hypertension were among the most frequently observed groups (22%, 21%, and 21%, respectively). Hip/knee problems (13%) and cancers (6%) were also clinical conditions of interest (Table 1). By geographical distribution, Bangkok the capital was the most studied area (28.8%) and the Southern region was the least studied area (15.2%).

**Table 1:** Population groups studied in Thai physical activity and sedentary behaviour research

Population groups	No. of studies
Non-clinical populations	
Students	136
General (no specific characteristics)	135
Occupation-specific populations	31
Groups based on PA/SB participation	27
Health-care students	27
Health-care professionals	20
Obese/overweight	18
Pre/post-menopausal women	14
Employees of a specific organization	10
Multiple populations groups	10
Pregnant and postpartum women	6
Ageing population	5
Religious groups	4
Smokers/non-smokers/ex-smokers	2
Others	19
Total	464
Clinical populations (general characteristic)	
Cardiovascular disease	22
Diabetes	21
Hypertension	21
Hip/knee injury/condition	13
Cancer	6
Respiratory disease/condition	4
Parkinson’s disease	3
Diabetes and hypertension	2
Epilepsy	2
Dementia	1
Total	100

### Measures of physical activity and sedentary behaviour

Out of 526 studies that investigated PA, most relied on self-reports only (73.4%) and 2.1% used both self-report and device-based measures. In nearly all of these studies (97.2%) PA was assessed using self-reported or proxy-reported questionnaires, and in most cases it was not specified which questionnaire or questionnaire item(s) were used for this purpose. The Global Physical Activity Questionnaire (GPAQ) and the International Physical Activity Questionnaire (IPAQ) were used in 25 (6.5%) and 23 studies (6%), respectively. Other self-reports were PA diary and logbook used in 14 studies (3.5%). Device-based measurement was used in 23 studies (4.4%), with accelerometer (*n* = 10) and pedometer (*n* = 9) being the most common devices. A large proportion of PA studies focused on exercise only (49.6%) or on total PA (32.5%). Domain-specific PA levels, including leisure-time, household, work-related, and transport PA, were examined in isolation in 2.5% of all PA studies. The most commonly studied domain of PA was leisure time (*n* = 16). Walking, as a type-specific PA, was investigated independently in 5 studies. In total, 5.9% of studies assessed a combination of domain- and type-specific PA levels, including exercise, sport and walking.

A total of 113 studies examined SB. Questionnaires were the most common measure of SB (91.2%), followed by activity diaries (4.4%), and device-based tools (3.5%). Most studies (65.5%) did not specify which questionnaires they used. GPAQ, IPAQ, and accelerometers were used in eight, four, and two studies, respectively. Screen time - including TV viewing, computer use, videogames, and internet/social networking - was the most commonly investigated type-specific SB (59.3%). Total sedentary or sitting time was assessed in 37 studies (32.7%), while SB in work and leisure-time domains was assessed in seven and five studies respectively.

### Study topics

Correlates of PA and/or SB were the most common topic and were investigated in 58.9% of studies. We identified 11 groups of PA/SB correlates. The most common were: socio-demographic correlates, such as, age, gender, and education level (24%); psychological correlates, such as mental health and well-being, self-efficacy, social behaviours, and cognitive tasks (20.9%); physical health and functioning correlates including physiological and biological functions, diseases, and health problems (19.8%); and social and cultural correlates, such as social support, beliefs, and social practices (11.4%). Other reported correlates included: health behaviours and lifestyles; physical environment; general health; physical skills, abilities, and fitness; academic performance; knowledge; and policy (Table 2).

**Table 2:** Number of studies investigating correlates and outcomes of physical activity and sedentary behaviour in Thai populations

Categories	Correlates		Outcomes	
	No. of studies	%	No. of studies	%
Socio-demographic	162	24	-	-
General health	37	5.5	9	4.2
Physical health and functioning	134	19.8	73	33.8
Physical skills, abilities, and fitness	10	1.5	42	19.4
Psychological	141	20.9	47	21.8
Health behaviours and lifestyle	35	5.2	32	14.8
Social and culture	77	11.4	2	0.9
Physical environment	38	5.6	1	0.5
Academic/school performance	8	1.2	-	-
Mortality	-	-	2	0.9
Knowledge	27	4.0	8	3.7
Policy	6	0.9	-	-
Total*	675	100	216	100

Note: *Multiple correlates and/or outcomes were investigated in some studies; hence the sum of the totals is greater than the total number of included studies

In total, 125 (22.2%) of the selected studies examined outcomes of PA and/or SB. Most of these studies examined physical health and functioning (33.8%), psychological outcomes (21.8%), physical skills, abilities, and fitness (19.4%), and health behaviours and lifestyles (14.8%). Other reported outcomes included general health; mortality; social characteristics; environmental characteristics; and knowledge (Table 2).

A number of measures were tested for validity and reliability in the Thai context (6.7%). These were mostly questionnaires (92.1%) such as GPAQ, IPAQ (short version), Godin-Shephard Leisure-Time Physical Activity Questionnaire (GSLTPAQ), Modifiable Activity Questionnaire for Adolescents (MAQA), and Perceived Benefits to Physical Activity Scale (PBEPAS). Two studies evaluated measurement properties of device-based measures of PA (pedometer and heart rate monitors). In one study [159] the Compendium of Physical Activities [599] was translated and validated.

## Discussion

This study is the first systematic scoping review that summarises current evidence of Thai PA and SB research to support national directions in promoting healthy lifestyle through PA. We identified a large number of PA and SB studies conducted in Thailand, covering a broad range of topics, and using a variety of study designs. There was an increase in the number of Thai PA and SB studies published per year, from one study in 1987 to 64 studies in 2015 (the search was conducted up to September 2016), indicating a growing interest in this research area.

The first Thai publication focusing on PA that we identified was a doctoral thesis from 1987 [289], however the vast majority of PA studies were published in the last two decades. Importantly, the number of Thai papers on PA published per year has been increasing (Fig. 2), indicating that this area of research is developing. It is important to note that half of the studies on PA focused on exercise only, overlooking other types of PA (such as occupational PA, household PA, transport-related PA, and leisure-time PA other than exercise). Historically, the terms ‘physical activity’ and ‘exercise’ have been used interchangeably, and exercise has been one of the most commonly studied types of PA [600]. However, exercise is only one out of several various specific types of PA that may be important for health. From the public health perspective, it is important to study not only exercise but also other types of PA. In Thailand, the term “exercise” had been more widely used until the “physical activity” term was formally promoted in 2002, when the national focal point was changed from the Exercise Unit to the Division of Physical Activity and Health [17].

This finding for Thai studies is consistent with global trends in PA research over the last few decades. The proportion of studies using total MVPA (and not just exercise) as a measure of PA has increased in the last decade [49, 67, 230, 231, 432]. To align with Thai national recommendations on total MVPA, this trend in gathering evidence should be continued in future studies. Importantly, we did not locate any Thai population-based study that considered participation in muscle-strengthening activities, which is similar to the situation in most other countries [601, 602]. Given that Thai national PA guidelines for adults include a separate recommendation on participation in muscle-strengthening activities [603], this suggests more studies on this specific type of PA are needed.

Up until the present, studies on SB in Thailand were less represented than those on PA. SB research is a more recent field of inquiry, compared with PA epidemiology. It has only been in the past two decades that SB has been recognised as a risk factor independent of PA level [604–607]. It was therefore expected that in Thailand SB research would be less developed than PA research. Of the 113 studies addressing SB, 40 looked at specific types of SB, such as TV viewing, computer/internet use, and playing video games. The earliest Thai study we identified that examined type-specific SB, was conducted in 1994, as part of a doctoral thesis focusing on TV viewing and academic achievement [99]. The first study assessing total SB was conducted in 2000, again as part of a doctoral thesis [434]. Since then, there has been a steady increase in the number of Thai papers on SB published per year, indicating an increasing recognition of the importance of this area of research. Given the prevalence of SB and its potential negative health outcomes [6, 12], it is important that future studies continue to focus on SB in Thai populations.

Recent methodological developments have led to the establishment of a new discipline, called time-use epidemiology, where periods of time spent in PA, SB and sleep are no longer considered as independent risk factors, but instead are treated as mutually exclusive and exhaustive parts of the 24-hour day [7–9]. The new approach allows for drawing conclusions about how different reallocations of time between PA, SB and sleep affect health, and for finding the optimal balance of these components of time-use for good health [9, 608]. In line with the new developments and with the public health guidelines adopted in other countries [609–611], the most recent Thai guidelines on movement/non-movement behaviours included recommendations on PA, SB, and sleep [603]. However, the current review found no Thai studies aligned with this new approach, suggesting that this might be an area worth exploring in future epidemiological studies in Thailand.

Almost 70% of all included studies (PA and SB) used cross-sectional designs, whilst the evidence base on determinants and outcomes of PA/SB from longitudinal studies and intervention trials is less developed, potentially due to affordability-related reasons. However, a limitation of cross-sectional data is that they do not allow to draw conclusions about the direction of analysed relationships. To get a better insight into potential causes and consequences of PA and SB, longitudinal studies and controlled intervention trials are needed. Most studies in Thailand assessed PA and/or SB using self-reports. Despite the limitations of self-report instruments [612], these are still the predominant measure of PA and SB in population-based surveys internationally [613, 614]. The use of device-based measures of PA and SB, such as accelerometers, in large-scale epidemiological studies is becoming more affordable, especially in high-income countries [615–617]. However, device-based measurement of PA and/or SB was seldom used in the Thai context. This is likely due to issues related to the high cost and participant burden associated with device-based measurement of PA and SB [614]. Although device-based measuring has limitations in assessing domain- and type-specific PA and SB levels, it may provide some data that cannot be reliably assessed by existing questionnaires (e.g. timing of different activities during a day, detailed data on weekly distribution of PA). To better understand patterns of PA and SB in Thai populations, future research might benefit from employing device-based measures alongside self-report measures.

Although studies included in this review used a variety of sampling methods and a broad range of sample sizes, few were conducted in large-scale population-representative samples. Besides national surveys funded by the Thai government using large scale data samples, such as National Health Examination Survey, Thailand Physical Activity Children Survey, National Physical Activity and Obesity Survey, and Health and Welfare Survey, 10 other studies also utilized a large scale sample (*n* range: 24,743 – 87,143) from the Sukhothai Thammathirat Open University cohort. To improve the generalisability of findings from observational studies, the use of such large, nationally representative samples should be encouraged in future Thai PA and SB research.

Across age categories, young to middle aged adults (18-59 years) were the most commonly studied population group, followed by older adults (60+ years). The convenience of conducting research among adults and older adults, compared with research among children and adolescents, in terms of ethical considerations, ease of access to participants, and simplicity of measurement, may partially explain why most Thai PA and SB studies focused on these age groups. Another reason may be that adulthood and older age are more convenient stages to observe health impacts of PA and SB, as symptoms of many diseases rarely occur in younger population groups [618]. However, in addition to a number of topics in PA and SB research that are specific for children and adolescent populations (e.g. levels and patterns of school-based PA and SB, tracking of PA and SB from childhood to adolescence, association of PA and SB with educational outcomes in primary and secondary schools, effectiveness of PA and SB interventions in the school setting), findings among adults may not be generalizable to the populations of children and adolescents, which calls for more studies of these age groups in the future.

Thai PA and SB studies covered a wide range of topics, largely consistent with PA/SB research trends in middle- and high-income countries globally [5, 10, 12, 27]. However, there has been limited research on environmental correlates/determinants of PA and SB, associations between PA/SB and mortality outcomes, PA/SB policy research, and validation of device-based measures of PA/SB in different Thai population groups (e.g. across different sociodemographic groups). Around one-third of Thai PA/SB papers were published in the Thai language, while the remaining papers were published in English. Publications in English have higher visibility in the international scholarly context. Alternatively, publications in Thai may better inform local public health stakeholders, media and the general non-academic readership. Ideally, all publications would be in both languages, but in reality this is not feasible. It is, therefore, important to keep a balance between publishing in Thai and English, by always carefully considering the primary purpose of the paper and the targeted readership.

This systematic scoping review has several strengths. First, a systematic search and study selection strategy were applied to identify eligible studies. Comprehensiveness of the search was achieved by using a large number of relevant PA- and SB-related keywords, conducting primary search through 10 bibliographic databases, and supplementing this with an extensive secondary search. Second, data on 39 variables were extracted from the selected studies, which allowed for a detailed interpretation of the current situation in Thai PA and SB research. Last, a key strength was that, since both Thai and English language papers were included, we were able to review a large number of studies that might not have been captured if we only reviewed papers in one language.

This scoping review has some limitations. Although we tried to identify as many studies as possible, we may have missed some studies because they were not indexed in the selected databases. Furthermore, given the large total number of included studies, we focused on providing general recommendations, whilst an in-depth assessment of each individual study was not feasible. Future reviews are needed to summarise findings on specific topics in PA/SB epidemiology within the Thai context, particularly by different age groups (e.g. children, adolescents, adults, and older adults.

### Summary recommendations for future research

Based on this systematic scoping review, it can be concluded that the greatest Thai PA/SB research gaps and limitations are: the lack of studies on SB; the use of unspecified and non-validated measures of PA and SB; a limited number of longitudinal studies; a limited number of studies conducted in population-representative samples; a limited number of studies conducted among children and adolescents; a limited coverage of several important PA/SB research topics, such as environmental factors. To provide stronger evidence and further improve the evidence base on PA and SB, future studies may consider several recommendations stemming from this review. First, given that SB research is less developed in the Thai context and that SB is emerging as a new and important health-risk factor among the Thai population [16], more studies on determinants of, outcomes of, and ways to reduce SB in the Thai population are needed. Future studies in Thailand would also be strengthened by using validated device-based and self-report measures of PA and SB. For a better understanding of determinants and outcomes of PA and SB in Thailand, future studies should aim to use longitudinal study designs. Additionally, to allow for better generalisation, more studies should use large, population-representative samples. Besides, future studies are needed specifically focusing on topics relevant to children and adolescents. Finally, research shows that PA is influenced by a number of individual, social, environmental, and policy factors [27, 619]. Whilst socio-demographic, psychological, and social correlates have been the topic of a number of Thai studies, more research is needed on environmental and policy-related correlates of PA and SB in Thailand.

## Conclusions

Thai research on PA and SB has rapidly evolved and received increasing attention in the last two decades. Substantial literature was mapped in this review, showing that existing research has a great potential to support the development of healthy lifestyles by increasing PA and reducing SB in Thailand. However, current evidence could be strengthened, particularly by conducting more research on SB, using sound research methods, and covering the full range of research topics on determinants and outcomes of PA and SB. By following the recommendations provided in this systematic scoping review, future studies may provide even stronger evidence needed to inform public health efforts to promote PA and reduce SB in Thailand.

## Additional files

Additional file 1: Search keywords. Detailed search keywords including the full search syntaxes used for each database. (PDF 183 kb)

Additional file 2: Data extraction table. The detailed table of all data extracted from each study included in this review. (XLSX 245 kb)

## Abbreviations

GPAQ: Global Physical Activity Questionnaire
GSLTPAQ: Godin-Shephard Leisure-Time Physical Activity Questionnaire
IPAQ: International Physical Activity Questionnaire
MAQA: Modifiable Activity Questionnaire for Adolescents
MVPA: Moderate-to-Vigorous Physical Activity
NCDs: Non-Communicable Diseases
NDLTD: Networked Digital Library of Theses and Dissertations
PA: Physical Activity
PBEPAS: Perceived Benefits to Physical Activity Scale
SB: Sedentary Behaviour
WHO: World Health Organization
