# Supplementary material for: Physical activity and sedentary behaviour research in Thailand: a systematic scoping review
Source: BMC Public Health. 2018 Jun 14;18:733. doi: 10.1186/s12889-018-5643-y (PMC6001063; doi:10.1186/s12889-018-5643-y)
Supplement: Supplementary file 1 — Search keywords. Detailed search keywords including the full search syntaxes used for each database. (PDF 183 kb) [file 12889_2018_5643_MOESM1_ESM.pdf]

Additional file – Search keywords

Search keywords used for physical activity and sedentary behaviour:

- *"physical activity"*
- *"physical inactivity"*
- *"physically inactive"*
- *“physical fitness”*
- *"energy expenditure"*
- *Exercise*
- *Sport*
- *Gym*
- *"motor activity"*
- *Walking*
- *Cycling*
- *Stair**
- *“active travel”*
- *“active transport”*
- *sedentar**
- *sitting*
- *"watching TV"*
- *"TV watching"*
- *"TV viewing"*
- *Television*
- *"video watching"*
- *"watching video"*
- *"computer use"*
- *"internet use"*
- *Gaming*
- *"video games"*
- *"social media"*
- *"screen time"*
- *Lifestyle*
- *"strength training"*
- *"resistance training"*
- *"weight training"*
- *"weight lifting"*
- *"muscle strengthening"*
- *"muscular strengthening"*
- *"muscle training"*
- *"muscle toning"*
- *"weight bearing training"*
- *"weight bearing strengthening"*
- *"strength or toning"*

- *"strength/toning"*
- *"strength / toning"*
- *"strength and toning"*

These keywords were combined with the following keyword for Thailand setting:

- *thai**

Search syntaxes:

Scopus:

title-abs-key("physical activity" OR "physical inactivity" OR "physically inactive" OR “physical fitness” OR "energy expenditure" OR exercise OR sport OR gym OR "motor activity" OR walking OR cycling OR stair OR “active travel” OR “active transport” OR sedentar* OR sitting OR "watching TV" OR "TV watching" OR "TV viewing" OR television OR "video watching" OR "watching video" OR "computer use" OR "internet use" OR gaming OR "video games" OR "social media" OR "screen time" OR lifestyle OR "strength training" OR "resistance training" OR "weight training" OR "weight lifting" OR "muscle strengthening" OR "muscular strengthening" OR "muscle training" OR "muscle toning" OR "weight bearing training" OR "weight bearing strengthening" OR "strength or toning" OR "strength/toning" OR "strength / toning" OR "strength and toning") AND title-abs-key(thai*)*

Pubmed

((("physical activity"[Title/Abstract] OR "physical inactivity"[Title/Abstract] OR "physically inactive"[Title/Abstract] OR “physical fitness” [Title/Abstract] OR "energy expenditure"[Title/Abstract] OR exercise[Title/Abstract] OR sport[Title/Abstract] OR gym[Title/Abstract] OR "motor activity"[Title/Abstract] OR walking[Title/Abstract] OR cycling[Title/Abstract] OR stair[Title/Abstract] OR “active travel”[Title/Abstract] OR “active transport”[Title/Abstract] OR sedentar*[Title/Abstract] OR sitting[Title/Abstract] OR "watching TV"[Title/Abstract] OR "TV watching"[Title/Abstract] OR "TV viewing"[Title/Abstract] OR television[Title/Abstract] OR "video watching"[Title/Abstract] OR "watching video"[Title/Abstract] OR "computer use"[Title/Abstract] OR "internet use"[Title/Abstract] OR gaming[Title/Abstract] OR "video games"[Title/Abstract] OR "social media"[Title/Abstract] OR "screen time"[Title/Abstract] OR lifestyle[Title/Abstract] OR "strength training"[Title/Abstract] OR "resistance training"[Title/Abstract] OR "weight training"[Title/Abstract] OR "weight lifting"[Title/Abstract] OR "muscle strengthening"[Title/Abstract] OR "muscular strengthening"[Title/Abstract] OR "muscle training"[Title/Abstract] OR "muscle toning"[Title/Abstract] OR "weight bearing training"[Title/Abstract] OR "weight bearing strengthening"[Title/Abstract] OR "strength or toning"[Title/Abstract] OR "strength/toning"[Title/Abstract] OR "strength / toning"[Title/Abstract] OR "strength and toning"[Title/Abstract]) OR ("physical activity"[MeSH Terms] OR "physical inactivity"[MeSH Terms] OR "physically inactive"[MeSH Terms] OR "energy*

expenditure"[MeSH Terms] OR exercise[MeSH Terms] OR sport[MeSH Terms] OR "motor activity"[MeSH Terms] OR sedentar[MeSH Terms] OR sitting[MeSH Terms] OR "watching TV"[MeSH Terms] OR "TV watching"[MeSH Terms] OR "TV viewing"[MeSH Terms] OR television[MeSH Terms] OR "video watching"[MeSH Terms] OR "watching video"[MeSH Terms] OR "computer use"[MeSH Terms] OR "internet use"[MeSH Terms] OR gaming[MeSH Terms] OR "video games"[MeSH Terms] OR "social media"[MeSH Terms] OR "screen time"[MeSH Terms] OR lifestyle[MeSH Terms] OR "strength training"[MeSH Terms] OR "resistance training"[MeSH Terms] OR "weight training"[MeSH Terms] OR "weight lifting"[MeSH Terms] OR "muscle strengthening"[MeSH Terms] OR "muscular strengthening"[MeSH Terms] OR "muscle training"[MeSH Terms] OR "muscle toning"[MeSH Terms] OR "weight bearing training"[MeSH Terms] OR "weight bearing strengthening"[MeSH Terms] OR "strength or toning"[MeSH Terms] OR "strength/toning"[MeSH Terms] OR "strength / toning"[MeSH Terms] OR "strength and toning"[MeSH Terms]) OR ("physical activity"[Other Term] OR "physical inactivity"[Other Term] OR "physically inactive"[Other Term] OR "energy expenditure"[Other Term] OR exercise[Other Term] OR sport[Other Term] OR "motor activity"[Other Term] OR sedentar*[Other Term] OR sitting[Other Term] OR "watching TV"[Other Term] OR "TV watching"[Other Term] OR "TV viewing"[Other Term] OR television[Other Term] OR "video watching"[Other Term] OR "watching video"[Other Term] OR "computer use"[Other Term] OR "internet use"[Other Term] OR gaming[Other Term] OR "video games"[Other Term] OR "social media"[Other Term] OR "screen time"[Other Term] OR lifestyle[Other Term] OR "strength training"[Other Term] OR "resistance training"[Other Term] OR "weight training"[Other Term] OR "weight lifting"[Other Term] OR "muscle strengthening"[Other Term] OR "muscular strengthening"[Other Term] OR "muscle training"[Other Term] OR "muscle toning"[Other Term] OR "weight bearing training"[Other Term] OR "weight bearing strengthening"[Other Term] OR "strength or toning"[Other Term] OR "strength/toning"[Other Term] OR "strength / toning"[Other Term] OR "strength and toning"[Other Term])) AND ((thai*[Title/Abstract]) OR (thai*[MeSH Terms]) OR (thai*[Other Term])))*

<u>Web of Science</u>

("physical activity" OR "physical inactivity" OR "physically inactive" OR "physical fitness" OR "energy expenditure" OR exercise OR sport OR gym OR "motor activity" OR walking OR cycling OR stair OR "active travel" OR "active transport" OR sedentar* OR sitting OR "watching TV" OR "TV watching" OR "TV viewing" OR television OR "video watching" OR "watching video" OR "computer use" OR "internet use" OR gaming OR "video games" OR "social media" OR "screen time" OR lifestyle OR "strength training" OR "resistance training" OR "weight training" OR "weight lifting" OR "muscle strengthening" OR "muscular strengthening" OR "muscle training" OR "muscle toning" OR "weight bearing training" OR "weight bearing strengthening" OR*

*"strength or toning" OR "strength/toning" OR "strength / toning" OR "strength and toning") AND thai**

<u>Other databases (through EBSCOhost)</u>

("physical activity" OR "physical inactivity" OR "physically inactive" OR “physical fitness” OR "energy expenditure" OR exercise OR sport OR gym OR "motor activity" OR walking OR cycling OR stair OR “active travel” OR “active transport” OR sedentar* OR sitting OR "watching TV" OR "TV watching" OR "TV viewing" OR television OR "video watching" OR "watching video" OR "computer use" OR "internet use" OR gaming OR "video games" OR "social media" OR "screen time" OR lifestyle OR "strength training" OR "resistance training" OR "weight training" OR "weight lifting" OR "muscle strengthening" OR "muscular strengthening" OR "muscle training" OR "muscle toning" OR "weight bearing training" OR "weight bearing strengthening" OR "strength or toning" OR "strength/toning" OR "strength / toning" OR "strength and toning") AND thai**
